# Form and functioning: contextualising the start of the global financing facility policy processes in Burkina Faso

**DOI:** 10.1080/16549716.2024.2360702

**Published:** 2024-06-24

**Authors:** Joël Arthur Kiendrébéogo, Orokia Sory, Issa Kaboré, Yamba Kafando, Meghan Bruce Kumar, Asha S. George

**Affiliations:** a Department of Public Health, University Joseph Ki-Zerbo, Ouagadougou, Burkina Faso; b Department of Research, Expertise and Capacity Building, Recherche pour la santé et le développement (RESADE), Ouagadougou, Burkina Faso; c Operations Division, Recherche pour la santé et le développement (RESADE), Ouagadougou, Burkina Faso; d Heidelberg Institute of Global Health, Medical Faculty and University Hospital, Heidelberg University, Heidelberg, Germany; e Institute of Tropical Medicine, Department of Public Health, Antwerp, Belgium; f London School of Hygiene and Tropical Medicine, Faculty of Public Health and Policy, London, UK; g Department of Health Systems and Research Ethics, KEMRI-Wellcome Trust Programme, Nairobi, Kenya; h School of Public Health, University of the Western Cape, Cape Town, South Africa; i Health Systems Extra-Mural Unit, South African Medical Research Council, Cape Town, South Africa; j Northumbria University, Department of Nursing, Midwifery and Health, Newcastle upon Tyne, UK

**Keywords:** Global Financing Facility for Women, Children and Adolescents: Examining National Priorities, Processes and Investments, Burkina Faso, global financing facility, health financing, external financing, policy analysis

## Abstract

**Background:**

Burkina Faso joined the Global Financing Facility for Women, Children and Adolescents (GFF) in 2017 to address persistent gaps in funding for reproductive, maternal, newborn, child, and adolescent health and nutrition (RMNCAH-N). Few empirical papers deal with how global funding mechanisms, and specifically GFF, support resource mobilisation for health nationally.

**Objective:**

This study describes the policy processes of developing the GFF planning documents (the Investment Case and Project Appraisal Document) in Burkina Faso.

**Methods:**

We conducted an exploratory qualitative policy analysis. Data collection included document review (*N* = 74) and in-depth semi-structured interviews (*N* = 23). Data were analysed based on the components of the health policy triangle.

**Results:**

There was strong national political support to RMNCAH-N interventions, and the process of drawing up the investment case (IC) and the project appraisal document was inclusive and multi-sectoral. Despite high-level policy commitments, subsequent implementation of the World Bank project, including the GFF contribution, was perceived by respondents as challenging, even after the project restructuring process occurred. These challenges were due to ongoing policy fragmentation for RMNCAH-N, navigation of differing procedures and perspectives between stakeholders in the setting up of the work, overcoming misunderstandings about the nature of the GFF, and weak institutional anchoring of the IC. Insecurity and political instability also contributed to observed delays and difficulties in implementing the commitments agreed upon. To tackle these issues, transformational and distributive leaderships should be promoted and made effective.

**Conclusions:**

Few studies have examined national policy processes linked to the GFF or other global health initiatives. This kind of research is needed to better understand the range of challenges in aligning donor and national priorities encountered across diverse health systems contexts. This study may stimulate others to ensure that the GFF and other global health initiatives respond to local needs and policy environments for better implementation.

## Paper context

- **Main findings**: There was a high level of political commitment to the Global Financing Facility in Burkina Faso, but its implementation has been hindered by policy fragmentation, competing interests, weak institutional anchoring, and misunderstandings.
- **Added knowledge**: This study documents the initiation of a global health initiative, specifically the Global Financing Facility, including the development and implementation of its planning documents, namely the Investment Case and Project Appraisal Document.
- **Global health impact for policy and action**: An understanding of the factors that facilitated or impeded the policy processes of developing and implementing the Global Financing Facility can inform the design and implementation of future initiatives.

## Background

Burkina Faso has made considerable progress in maternal and child health over the last thirty years, even though targets 4 and 5 of the Millennium Development Goals were not met. Research on reproductive, maternal, newborn, child, and adolescent health and nutrition (RMNCAH-N) details multifaceted challenges and weaknesses, related, for example, to health care services users’ level of education and income [1–4], geographical access [5,6], provision of quality care [2,4,7] and socio-cultural constraints such as ethnicity, gender disparities, marital status and residence [1,5,8]. It is within this context, that in September 2017, Burkina Faso joined the Global Financing Facility for Women, Children and Adolescents (GFF) to mobilize resources to address persistent gaps in RMNCAH-N [9].

The GFF is meant to act as a catalyst for domestic and external funding sources to ensure adequate funding for RMNCAH-N [10,11]. While GFF contributors are varied [11,12] – hence the name ‘GFF Multi-donor Trust Fund’, the GFF is hosted by the World Bank (WB). Specifically, it is functionally linked in each country as a top up to the amount of funding available from the WB’s International Development Association and International Bank for Reconstruction and Development [10]. Beyond funding, the GFF also seeks to better coordinate and streamline RMNCAH-N investments in recipient countries [11,12], in the following ways: i) encourage the development of a government-led sustainable multi-stakeholder engagement platform, ii) identify and support priority financing and systems reforms, iii) help implement functional, real-time national data platforms, iv) support the development of a prioritized and costed Investment Case (IC) [13].

Burkina Faso’s participation in the GFF is consistent with its efforts to achieve Sustainable Development Goals 2 and 3, targeting resources and attention to marginalised groups and RMNCAH-N’s priority interventions to yield greater impact and equity [14]. As a new global funding mechanism involving many national and international players with different histories and interests, operationalizing the GFF is not without challenges [15], as is the case for prior global health initiatives [16–18]. This paper describes the policy processes that supported the development and restructuring of the GFF planning documents, i.e. the IC and the Project Appraisal Document (PAD) in Burkina Faso. Our objective is to inform future GFF processes and those of other global funding initiatives to ensure further learning so that they deliver on their promises. The study was done as part of the ‘Countdown GFF policy analysis collaboration’ which examines the early days of the GFF through policy content and country case studies [19].

## Methods

We conducted a descriptive, exploratory qualitative case study. Case study methodology allows the exploration and understanding of complex phenomena in their context [20]. We adapted Walt and Gilson’s health policy triangle to guide our work and made a preliminary analysis of our data [19], which we then grouped into three main inductive themes: a strong focus on RMNCAH-N despite fragmented policy; conflicting interests and misunderstandings; and a weak institutional base for the IC.

### Study setting

Burkina Faso is a low-income Sahelian country in West Africa with a size of about 274,200 km^2^. Its economy is fragile, relying largely on agriculture, which is subject to the vagaries of the climate, and on exports of raw materials such as gold and cotton. Table 1 presents the country’s key demographic and RMNCAH-N indicators.

**Table 1.** Burkina Faso key demographic and RMNCAH-N indicators.

Key demographic and RMNCAH-N indicators	Value
Total population (in 2019)	20,505,155
Life expectancy at birth (years) (in 2019)	61.9
Maternal mortality ratio (per 100,000 live births) (in 2020)	264
Under 5 mortality ratio (per 1,000 live births) (in 2021)	83
Neonatal mortality ratio (per 1,000 live births) (in 2021)	25
Urbanization rate (%) (in 2019)	26.1
Population under 15 years of age (%) (in 2019)	45.3
Total fertility rate per woman (in 2019)	5.4
Adolescent girls’ fertility rate 15–19 years (‰) (in 2019)	67.8
Unmet need for contraception (% of married women ages 15–49) (in 2021)	22.0
Pregnant women receiving prenatal care (%)	80.0
Births attended by skilled health staff (% of total) (in 2015)	80.0
Completeness of birth registration (%) (in 2010)	77.0
Adult literacy rate, by sex (% of people ages 15 and above) (in 2021)	Male = 54.5 Female = 37.8

Source: Burkina Faso’s General Population and Housing Census in 2019;^23^ World Bank Open Data;^24^ World Bank Gender Data Portal^25^.

Burkina Faso had a popular uprising on 30 October 2014, which ended the mandate of President Blaise Compaoré, who had been in power for 27 years [21]. Since then, the political and security situation has deteriorated, with numerous terrorist attacks and many internally displaced people (IDPs). As of July 2023, five presidents have succeeded Mr. Blaise Compaoré, and three military coups have taken place. The deleterious political and social context has led to dysfunctions in the health system, such as: frequent changes of Ministers and Secretaries General (a total of six Ministers and six Secretaries General between 2014 and 2023); closure of several health facilities; health care workers fleeing insecure zones; patient referral problems because of ambulance attacks; impairment of the quality of care caused by inadequate supplies of drugs, equipment and other inputs.

### Data collection and management

We used two methods for data collection: document review and in-depth semi-structured interviews. Data were collected from November 2022 to March 2023.

The document review included 74 documents in total, including official policy documents, grey literature, scientific publications and legal documents, as detailed in Table 2. Data extracted from these documents informed the timeline, process and context of events, the identification of priorities defined in the GFF documents and the mapping of key stakeholders and their roles [19].

**Table 2.** Documents reviewed.

Document type	n	Examples
Official policy	15	International (2) and national (11) policies, strategies and action plans related to RMNCAH-N, statistical yearbook (1), and census document (1)
Scientific data/evidence	18	Journal articles (5), published reports (12), policy-brief (1)
Grey literature	31	Activity reports/workshop minutes (11), memos (3), PowerPoint presentations (12), roadmap (1), concept/technical notes (4)
Legal/administrative text	7	Law (1), decrees (2), memorandums (2), cooperation agreements between government and donors (2)
Other	3	Terms of reference (1), press release (1), newspaper article (1), e-mail exchanges and website consultation (not counted)
**TOTAL**	**74**	

Stakeholder mapping identified an initial list of 70 actors involved at some point in the GFF document development and implementation. They included individuals from the Ministry of Health (MOH), the Ministry of Finance (MOF), the Ministry of Education, CSOs (Civil Society Organizations), NGOs (Non-Governmental Organizations), the private health sector, and technical and financial partners. From this initial list, 32 respondents were selected purposively, based on their level of involvement in the process and the ability to locate them. Of these, 23 were interviewed inclusive of GFF and WB personnel, government officials, civil society, and technical consultants (Table 3).

**Table 3.** List of key informants.

Respondent type	# Contacted	# Interviewed	%
GFF (Headquarters/National) and WB	8	6	75
Government (MOH, MOF, MOE)	20	15	75
Other (CSOs, Private Sector, Consultants)	4	2	50
**TOTAL**	**32**	**23**	**72**

In-depth interviews were conducted in French, remotely or face-to-face depending on the respondent’s preference, using a semi-structured guide. After providing consent, each interview lasted 45–60 minutes and covered the period from the start of the GFF in Burkina Faso in 2017 until the restructuring of the PAD in 2021. The recorded interviews were transcribed verbatim into Microsoft Word.

### Data analysis

The research team met weekly to reflect on the data collection and analysis process and co-developed the analysis tools. Document review and interviews were coded in NVivo software based on predefined themes. Two individuals did the coding separately (IK, OS) and then compared to ensure intercoder reliability [22]. Disagreements were discussed with two other members of the research team (JAK and YK) to reach a consensus. Specific quotes identified to support the results were translated to English by JAK.

### Positionality, reflexivity, and ethics consideration

This study received ethical approval from Burkina Faso’s Health Research Ethics Committee (deliberation N° 2022-07-166). All respondents gave voluntary and informed consent to participate in the study.

JAK worked as a short-term consultant for the WB from March 2017 to March 2019. In this capacity, he assisted in the development and writing of the PAD and actively supported Burkina Faso’s involvement with the GFF and the initial stages of drafting the IC. This ‘insider’ position was an advantage in accessing some respondents, certain grey literature, as well as reconstructing some facts and understanding certain relatively complex WB and GFF procedures. To minimize biasing the research, JAK did not participate in the interviews. Instead, he provided a critical eye in open discussions with the other research team members once they had carried out the first analyses and interpretations of the interview data. This allowed for a nuanced and less partisan perspective on the various elements or events reported in the study.

The preliminary results of the study were presented to representatives of the MOH and the GFF for review, and at the 15^th^ World Congress of the International Health Economics Association (IHEA) in July 2023. Feedback was received and incorporated.

The Countdown GFF policy analysis collaboration co-developed a ‘principles of an equitable partnership’ document to provide background on the collaboration, clarify roles and responsibilities, and document ways of working, including data governance and authorship principles to strive for equitable partnership in the collaboration (see Supplementary File 1 for more details).

## Results

**Figure 1.**
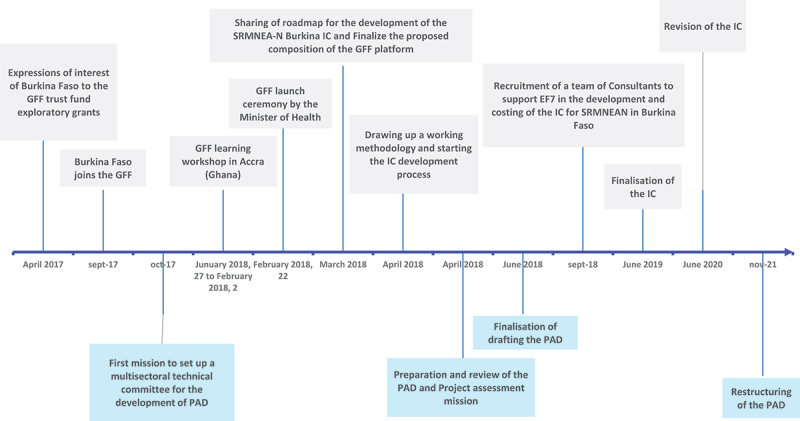
Timeline of key events in the PAD and IC development process in Burkina Faso.

Source: Authors

As mentioned earlier, the GFF engagement in Burkina Faso began in 2017 and development of the IC and PAD started in that year. The PAD was finalised in June 2018 due to WB approval deadlines, followed by the IC a year later. The PAD’s total budget was US$ 110 million, of which US$ 80 million came from IDA, US$ 20 million from the GFF Trust Fund and US$ 10 million from the Power of Nutrition Multi-Donor Trust Fund. The IC had a total planned budget of US$ 1,818 million with an estimated funding gap of 40% that was meant to be filled by mobilizing additional resources. The IC was subsequently revised in 2020, and the PAD was also restructured in 2021 largely responding to needs arising from the insecurity in the country (Supplementary File 2). Important milestones in the PAD and IC development process in Burkina Faso and their alignment with the national policy environment are presented in the timeline (Figure 1).

Three key themes emerged from the data analysis of the policy content, process, actors, and context examined: the prioritisation of RMNCAH-N despite policy fragmentation; negotiating different interests and misunderstandings; and the lack of institutional anchoring for the IC.

### Strong political prioritization of RMNCAH-N was undercut by fragmentation in financing and delivering on these goals

Recognizing the importance of RMNCAH-N, Burkina Faso’s government endorsed international goals for maternal and child health and supported these commitments, with policy actions including a payment waiver for antenatal care introduced in 2003, followed by a subsidy covering 80% of the direct medical costs of emergency obstetric and neonatal care between 2006 and 2015 [23,24], and a national user fees exemption policy for women and children under five from 2016 [25,26]. As such, Burkina Faso had strong political commitment to work with the GFF from its entry and even co-hosted the GFF replenishment event in November 2018 [27]. The replenishment was an opportunity for the Head of State, democratically elected in 2015 after the popular uprising [21] to affirm Burkina Faso’s commitment to improving its health and nutrition status [27].

Nonetheless, before and after joining the GFF, Burkina Faso had a complex policy environment with a long list of ‘competing’ policies, strategies, and plans related to RMNCAH-N, both within and outside the health sector (see Box 1 for examples). A plethora of implementing agencies and 26 national and international donors were working in the country using a variety of funding mechanisms according to a mapping carried out in 2018 [28]. The forecasted budget for RMNCAH-N in 2018 was USD$ 195 million, of which only 28.44% came from the State [28].

### Negotiating different interests and misunderstandings

**Figure 2.**
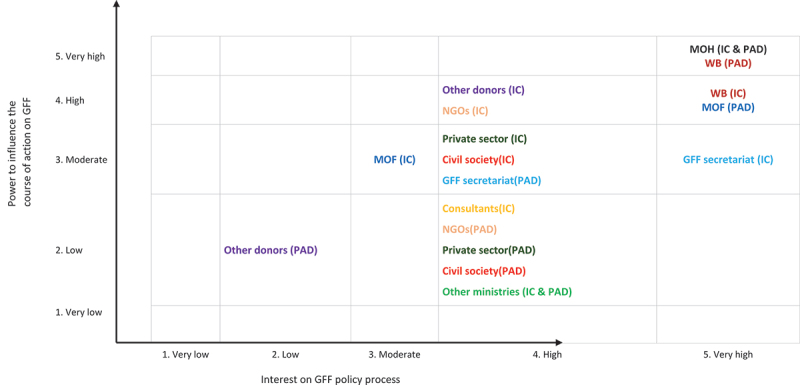
Stakeholders mapping in relation to their power and interest on the GFF policy process.

From the outset, the IC and PAD development processes were consultative, involving a range of national and international actors (Table 4 and Figure 2), convened under the leadership of the MOH (Family Welfare and Nutrition directorates) and the WB.

**Table:** 

**Box 1**: Examples of policies, strategies, and plans related to RMNCAH-N.
Integrated Strategic Plan for Reproductive, Maternal, Newborn, Child, Adolescent, Youth and Elderly 2017–2020 National Family Planning Acceleration Plan 2017–2020 & National Family Planning Plan 2021–2025 Strategic Plan for Under-5 Child Survival Strategic Plan for Nutrition 2010–2015 Plan to Promote the Scale-up of Infant and Young Child Feeding (IYCF) Best Practices 2011–2025 National Policy on Integrated Early Childhood Development Reproductive health policies and standards Plan for the elimination of mother-to-child transmission of HIV, syphilis, and hepatitis B 2021–2025 Strategic plan to strengthen paediatric HIV prevention and care National Gender Policy 2009–2019 Health sector strategic plan to promote the elimination of female genital mutilation 2019–2023 Investment case for the three transformative outcomes (zero unmet need for family planning; zero preventable maternal deaths; zero gender-based violence and harmful practices) National communication and advocacy strategy for nutrition 2020–2024 Strategic Health Plan for Youth and Adolescents 2022–2026 National multisectoral nutrition policy 2020–2029 Multi-sector nutrition strategic plan 2020–2029 Guidance document on the harmonisation and alignment of interventions following the ‘One Plan, One Budget, One Report’ approach in Sexual and Reproductive Health and Rights (SRHR) and Gender-Based Violence (GBV)

**Table 4.** Key players involved in preparing the IC and the PAD, along with their roles and the nature of their power.

Actors	Role	Nature of power
MOH	Technical lead and coordination of the overall process, ensure that priorities and activities are aligned with national policies and strategies	Bureaucratic (hierarchy and/or seniority) & technical
MOF	Technical orientations regarding budget procedures, resources negotiations and assistance for budgeting	Political & technical
Other Ministries	Identification of problems, priorities, and strategies; participation in discussions and experiences sharing	Technical
WB	Technical (advice, guidance) and financial support; drafting of the PAD	Financial & technical
GFF Secretariat	Technical (advice, guidance) and financial support	Financial & technical
Donors other than the WB (bilateral, multilateral)	Technical orientations, including their preferences for funding, and resources mobilization	Financial & technical
International NGOs	Technical orientations and resources mobilization	Financial & technical
Consultants	Costing and drafting of the IC according to the guidelines provided by the technical working groups and the Functional Team 7	Technical
Private sector	Identification of problems, priorities, and strategies; participation in discussions and experiences sharing	Technical
Civil society	Identification of problems, priorities, and strategies; participation in discussions and experiences sharing	Technical

Unique to Burkina Faso, the GFF national focal point was a former General Secretary of MOH, with good connections with key stakeholders. Even as General Secretary, he was perceived as more of a technocrat than a politician and therefore caused limited polarisation, contrary to what might be expected given the political connotations that the position can have. While interests and types of power varied across stakeholders, the technical, financial power and seniority of certain actors carried more weight than others. Indeed, the room for manoeuvre was sometimes limited because some funders had their own preferences and influenced the definition of priorities.

Some donors have put a great deal of effort into defending certain areas. They formed, with the support of the Ministry of Health departments, whose activities they financed, small advocacy groups which tried to influence certain aspects to be retained. A policymaker from the MOH [KI-PM15]

The WB team responsible for drawing up the PAD were pressed to finalise it by June 2018, so that it could be considered and examined by the WB’s Board of Directors before the end of the year [KI-D1]. The deadline was met by accelerating the consultation process with national stakeholders and speedily deciding on priorities, even when each stakeholder was often concerned with defending its own agenda.

There were two different teams who disagreed about how to operationalize strategic purchasing [under component 1 of the PAD]: the team advocating for results-based financing and the team backing free health care policy. The latter was opposed even to the concept of results-based financing. The two parties couldn’t even talk without emotion. A former WB staff [KI-D1]

The IC was also initially meant to be concluded in 2018. An existing platform within the MOH, the *Functional Team 7*, devised a roadmap in March 2018 with a dual purpose, namely ensuring that: i) the IC is ready by the end of September 2018 so that the Head of State can prepare to co-chair the conference on the replenishment of the GFF in Oslo on 5 and 6 November 2018 – this led to the recruitment of the three consultants to support the process; ii) the government takes ownership of the process – this prompted the setting up of four technical working groups (see Supplementary File 3 for more details on the GFF policy processes in Burkina Faso). However, the IC was released one year after the PAD, in June 2019. Two main factors explained this delay. The first one was the multi-sectoral approach adopted in drawing up the IC, which required time to establish contacts or to explain the GFF to new actors:

The challenge was multi-sectorality. It required a lot of consultation with other actors, other ministries. There were certain key players with whom we were already partners, but we realized that there were other players with whom we didn’t have much contact, and who also needed to be involved. So, it was still a challenge to get the other sectors involved, and that delayed certain aspects a bit. A policymaker from the MOH [KI-PM6]

We started working with a list of stakeholders that was as broad as possible, but which grew as more and more stakeholders were identified. There were at least thirty or forty participants, so it was a bit tricky because not everyone had the same level of information about the GFF. A policymaker from the MOH [KI-PM9]

The second one was the lack of understanding among certain players that not all the resources to finance the IC were immediately available and that additional funds still had to be mobilized to fill the funding gaps – even though ideally activities must be prioritized according to available funds. This had a negative impact on their motivation and enthusiasm at a certain point in the process.

We thought that the GFF was additional funding that would be distributed between the various entities, whether ministries, civil society, the private sector, etc. But it was only afterwards that we realized that it wasn’t, that the GFF was merging with an existing funding mechanism to stimulate prioritization at national level. An actor from the MOF [KI-PM1]

A lot of the actors who came to the GFF thought they were going to get funding […] So one day when one of the actors realized it was only US$20 million, he said he didn’t know why he was going to all this trouble. An actor from the MOF [KI-PM10]

### Implementation of the IC and PAD was not institutionally led or supported by all key stakeholders

It was not decided from the outset which department of the MOH should be responsible for carrying the IC forward. This created confusion about which authority within the MOH should coordinate and monitor IC activities, delaying the processes and leading to continued bypassing of the mechanism.

If you look at the journey of the investment case at the Ministry of Health, it says a lot. The institutional anchoring was not well defined. We didn’t know whether the investment case should be steered by a directorate-general, i.e. the general secretariat, or by a technical secretariat. There was a lot of uncertainty before it went to the Directorate General for Studies and Sectoral Statistics. A policymaker from the MOH [KI-13]

Subsequently, high turnover among leadership of MOH prevented sustained political attention to the IC and its adoption by all stakeholders, especially those who were not part of the process from the outset. As such, from a policy perspective the multiplicity of strategic documents continued and from the financing side, some donors continued to fund RMNCAH-N activities directly via health districts, NGOs and CSOs, without explicit reference to the IC.

How is it that, after drawing up an investment case with so many players, there are still donors approaching individual technical departments of the Ministry of Health to draw up yet more documents? Was the misunderstanding only on the part of the Ministry of Health? Did the donors misunderstand as well? Or did the donors just play a two-faced game, being there only to applaud but knowing that they wouldn’t use it? A policymaker from the MOH [KI-PM8]

I get the impression that there hasn’t been a good take-up because not everyone knows what to do with the investment case […]. This can also be explained by staff turnover. When I ask my colleagues what’s in the revised investment case, they don’t know. So, there’s a real lack of communication between departments. […] We’ve always worked based on our strategic plan; it was only recently, when we wanted to draw up the new plan, that we were told no, there’s the investment case, which is a document you must use. A policymaker from the MOH [KI-PM5]

The implementation of the PAD also ran into challenges. As of February 2021, the disbursement rate was 14% for the IDA funds and 30% for the GFF trust funds [29]. Several factors led to poor implementation. Firstly, administrative issues related to set up, recruitment and banking. Secondly, the turnover at the MOH and the MOF, as well as at the WB, and changes in the MOH’s organizational chart, caused poor ownership of the project including agreements previously reached between stakeholders. Thirdly, technical issues, notably the lack of consensus on how to implement strategic health purchasing, which formed the bulk of the PAD’s Component 1. As a result, a consensual operations manual could not be drawn up. These difficulties led to a restructuring of the PAD in November 2021, with its extension until June 2024.

## Discussion

This study looks at the policy processes involved in developing Burkina Faso’s GFF planning documents, namely its IC and PAD. Our findings show some commendable aspects to these processes, in line with the recommendations of the GFF secretariat, such as the use of an inclusive and multistakeholder approach led by countries willing to invest more and efficiently in RMNCAH-N, and an evidence-based prioritisation [11]. However, our findings also highlighted factors mainly related to the context and the process that led to problems in implementing the commitments made and that are important to consider for Burkina Faso as it moves forward.

Implementing the GFF is not an easy task [15] – some challenges related to this endeavour, consistent with our findings, have been pointed out by some authors critically reflecting on the GFF [10,12]. These, for example, include donors’ dominance over the choice and funding of priority interventions, the limited explicit use of the IC as a reference document to develop RMNCAH-N activities, and mobilizing and coordinating resources from various national and international donors to ensure effective funding of the IC. With regard to health policy in Burkina Faso specifically, Shearer et al. [30] observed that the country, which is heavily dependent on foreign aid, is very susceptible to the ideas of donors, which are sometimes driven by their own interests. All these factors hindered policy ownership and are not new to the broader development financing landscape [31,32], raising the issue of government leadership in determining and implementing public health policies [33–35].

Government leadership is a key input in the GFF logic model, which aims to be different from other multilateral support initiatives by improving country ownership, donor coordination and harmonisation, and by being participatory, inclusive, and results-oriented [36]. An important process outcome of this model would be for these features to be systematically pursued and to be present in the way other policies are designed and formulated. This would enable the GFF investment to have a measurable and sustained impact on national health policies. It could also demonstrate the initiative’s added value beyond the relatively small additional resources provided by the GFF Trust Fund, which seem to be low in relation to the effort and workload required to develop the IC. However, other initiatives similar to the GFF, which were also intended to bring about a paradigm shift in development aid, have largely failed [37]. There is, therefore, a need to continuously evaluate the implementation of the GFF in target countries to identify bottlenecks, quickly find solutions and draw lessons. Success would imply that the GFF model is well communicated and understood by all stakeholders, which in Burkina Faso did not always seem to be the case. For example, it is important for policy actors to distinguish between the process of engaging them and trying to attract external aid and domestic resources, and the Trust Fund itself, which is meant to be catalytic.

For the GFF to deliver on its promises, government leadership is crucial. But leadership itself is a multi-faceted concept [38], and of growing interest in the health field [39–42]. Chunharas and Davies [39] ‘consider leadership as the ability to identify priorities, set a vision, and mobilize the actors and resources needed to achieve them’. Several styles and levels of leadership have been described. For example, we have transformational leadership, where leaders create a vision, act on intrinsic motivation, and inspire subordinates or partners to go beyond expectations; versus transactional leadership, where extrinsic motivation is heavily relied on to achieve stated objectives, without further expectations [43,44]. Also, leadership is not the prerogative of individuals alone, it can permeate the entire health system operating at any level, the so-called distributed or interactive or collective leadership as opposed to command-and-control leadership [39,42,45]. The latter is easier to implement but not necessarily the most effective, especially in contexts such as Burkina Faso, which are characterised by high political instability and a high turnover of senior officials in the MOH. Each new leader brings a new vision and direction, making it sometimes necessary to repeat the planning process several times to adapt to political dynamics and ensure consensus moving forward.

Given the multiplicity of players and complexities within health systems, there is a need to transition from the command-and-control leadership to the transformational and distributed or interactive or collective leaderships. These types of leadership should be an intrinsic characteristic and a routine within health organizations and health systems to be sustained over time. Such leaderships seemed to have been lacking in Burkina Faso following the adoption of the GFF documents, resulting in bottlenecks in the PAD’s implementation and a relatively little consideration given to the IC. For example, such leaderships from the MOH could have leveraged the strong national political support for the GFF to maintain momentum in delivering activities and not letting it falter as witnessed. They would have helped the GFF process to be more resilient and not suffer from the high turnover of national and international players. Such leaderships would also have enabled administrative bottlenecks and disputes between stakeholders in the PAD’s preparation and implementation to be anticipated and/or managed quickly, through the emergence of a shared vision and collective action. They would have enabled all parties to reach the same understanding of some issues at stake and prevent disappointments that demotivated some – for example, understanding that GFF is not business as usual where money is readily made available to finance activities, but that ongoing efforts to mobilize both domestic and external resources are needed. These efforts could be integrated into a national health financing strategy to be developed, revised, or revitalized, which would help to streamline resources and reduce the fragmented financing of RMNCAH-N. Finally, such leaderships would also have to ensure at an early stage that the IC is properly anchored internally and genuinely serves as a reference document for RMNCAH-N, thereby allowing donors alignment and preventing plethora and fragmentation of policies and strategies as observed. Such leadership, however, may need capacity building and imply availability of adequate resources [46,47], as well as technical, political, diplomatic, anticipation, adaptability, and communication skills [39,42,45].

Transformational leadership promotes organizational learning, which in turn improves organizational performance [48,49]. Such leadership is also recognised as essential for universal health coverage, with the development of specific programmes to empower countries, although complex political and technical challenges prevent rapid results [50]. The ability of MOHs to become learning organisations, where information sharing, dialogue and collaboration are crucial, could help break down silos and foster the emergence of collective leadership [51,52].

One mechanism at the core of the GFF process, whose success also depends on strong leadership and governance, is its inclusive and multi-sectoral approach [53,54]. A multisectoral approach can be defined as a ‘deliberate collaboration among various stakeholder groups (e.g. government, civil society, and private sector) and sectors (e.g. health, environment, and economy) to jointly achieve a policy outcome’ [55]. This approach is strongly recommended because it enables to harness the different knowledge, expertise, standing, and resources from all these actors to account for the many determinants of health to improve policy and ultimately health outcomes [55–57]. The multi-sectoral approach was effective and much appreciated by respondents, although subsequent results from the PAD and the IC implementation revealed that it is not sufficient to trigger action and prevent blockages. In short, to deal with all the complexities intrinsic to health policy implementation, strong leadership and governance are key [54,57].

The fragile security situation and political instability have had a negative impact on the implementation of the IC and PAD. Specifically, the security situation puts Burkina Faso on the list of ‘Fragile and conflict-affected states’, with the risk that global actors (donors, NGOs, and humanitarian actors in general) strongly influence the choice of priorities, especially as the country is still learning to cope with these new challenges. The revision of the IC and the restructuring of the PAD have sought to reflect the impact of the security crisis, but Burkina Faso certainly has much to learn from other ‘Fragile and conflict-affected states’ about improving the resilience of its health system. The experience to be gained concerns all health system building blocks and domains, covering aspects as diverse as health financing [58–61], human resource for health [62–65], health services delivery [66–68], health system governance [69–71], state building [72,73] and health system strengthening more broadly [74,75], which still suffer from crises of this nature. The contribution of the GFF in this respect is important and desirable, and further specific reflections on the subject are welcome.

The insecurity and political instability in Burkina Faso may lead to greater centralisation of decision-making to ensure maximum control, and it is difficult to develop decentralized leadership in such a context. However, for multisectoral initiatives, command-and-control leadership would lead to bureaucracy and slower implementation. Therefore, even in such difficult situations, it is worth reflecting on the conditions for successful distributed leadership. In the case of the GFF, this would mean discussing the role that decentralized levels of government can play in the implementation leadership model. A multi-layered and multi-level leadership model across cross-sectoral dynamics may be required – an area that is largely unexplored.

### Study strengths and limitations

Being retrospective provided respondents the space to recall more directly on power and politics of the processes without fear of retribution. However, this introduces limitations related to memory or recall bias, as the negotiation process and the drafting of the GFF documents began in September 2017. Second, change in individual positionality and turnover affected the results: some identified actors were no longer at their positions and could not be found or had left Burkina Faso. Some national staff were recruited by donors, and this change of role and positionality may have introduced some bias into the responses provided. To minimize these biases, we diversified the people interviewed and strove to contact those we could. We also triangulated the interview data across respondents and with the document review. The insider positionality of JAK in the document development process also provided access to grey literature in the form of emails, meeting notes, etc. that were valuable for contextualizing the findings.

## Conclusions

The GFF is currently implemented in 36 countries, but exploratory studies like ours analysing the initiation and implementation of PADs and ICs have scarcely been carried out. Such studies are, however, essential for identifying and understanding factors that helped or hindered these processes, to take account of them in the future. Despite best intentions and extensive planning efforts, implementing these two documents has been problematic in Burkina Faso’s context. Factors involved in include policy fragmentation despite RMNCAH-N prioritization, the negotiation of different interests and misunderstandings, and institutional anchoring within the government. To address these challenges, we conclude that transformational and distributive leaderships are key to enabling further implementation of GFF planning documents for such reforms to have an impact.

## Data Availability

The datasets used and/or analysed in this study are available from the corresponding author on reasonable request. This paper is part of the *Global Health Action* Special Series, “Global Financing Facility for Women, Children and Adolescents: Examining National Priorities, Processes and Investments”, available in Volume 17-01.
